# Surface Analysis—From Crystal Structures to Particle Properties

**DOI:** 10.1021/acs.cgd.4c00259

**Published:** 2024-05-01

**Authors:** Alexandru A. Moldovan, Andrew G. P. Maloney

**Affiliations:** The Cambridge Crystallographic Data Centre, 12 Union Road, Cambridge CB2 1EZ, U.K.

## Abstract

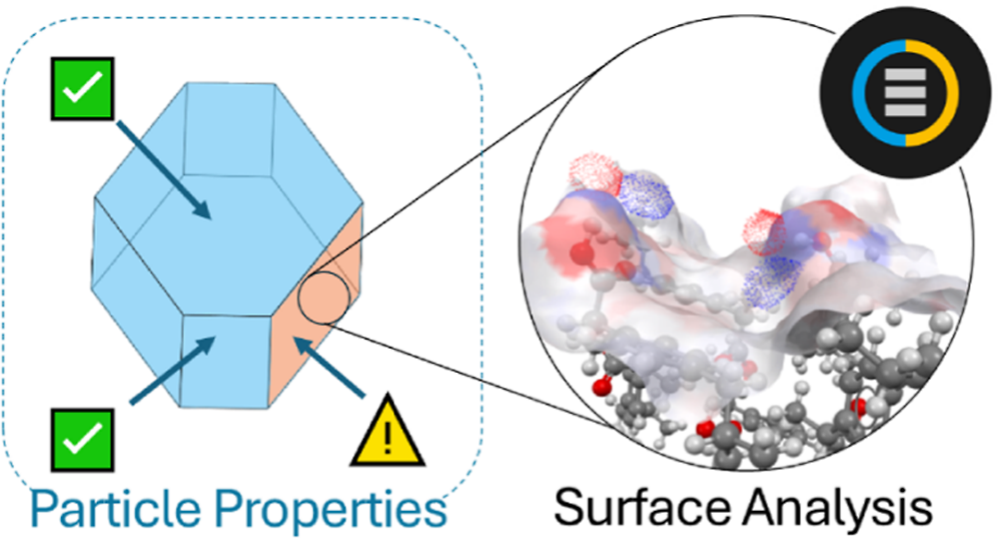

Understanding the surface properties of particles is crucial for optimizing the performance of formulated products in various industries. However, acquiring this understanding often requires expensive trial-and-error studies. Here, we present advanced surface analysis tools that enable the visualization and quantification of chemical and topological information derived from crystallographic data. By employing functional group analysis, roughness calculations, and statistical interaction data, we facilitate direct comparisons of surfaces. We further demonstrate the practicality of our approach by correlating the sticking propensity of distinct ibuprofen morphologies with surface and particle descriptors calculated from a single crystal structure. Our findings support and expand upon previous work, demonstrating that the presence of a carboxylic acid group on the {011} facet leads to significant differences in particle properties and explains the higher electrostatic potential observed in the block-like morphology. While our surface analysis tools are not intended to replace the importance of chemical intuition and expertise, they provide valuable insights for formulators and particle engineers, facilitating informed, data-driven decisions to mitigate formulation risks. This research represents a significant step toward a comprehensive understanding of particle surfaces and their impact on products.

## Abstract 2

We describe a new systematic and reproducible approach to predicting and approximating molecular-scale particle surfaces derived from experimental or calculated crystal structures. This approach enables visualization and analysis of surface chemistry and topology. The use of these tools are demonstrated through their application to understanding punch sticking for different particle shapes of ibuprofen.

## 1 Introduction

Particle properties significantly impact the performance of manufactured products, particularly in the formulated product industries, including fast-moving consumer goods, pharmaceuticals, agrochemicals, and dyes.^1−4^ The surface chemistry and roughness of particulates can affect processing qualities such as flow, hygroscopicity, packing, and sticking.^5−10^ Consequently, understanding particle properties is crucial in making formulation decisions, but such an understanding typically requires extensive trial-and-error studies, which can be costly.

Digital design tools have been used for formulation and product performance optimization. One such technique involves the use of molecular modeling to compute interactions between solvents and surfaces,^11,12^ as well as surface–surface interaction energies.^13,14^ This method provides a detailed understanding of the molecular-level behavior of materials in different conditions. Moreover, statistical techniques such as machine learning models can be employed to predict processing behaviors.^15^ These models are based on large data sets and can identify patterns and relationships between different variables.

However, it is essential to note that these methods often require significant computational effort and meticulous consideration of force fields. Specific techniques may be limited in their applicability and consequently may not provide a complete description of the material in terms of its chemical and physical attributes. Therefore, it is crucial to choose the most appropriate methodology based on the specific requirements of the material being formulated.

Prior work has described structural informatics-based approaches toward rational particle design and understanding, using simple computational descriptors to describe the properties of lamotrigine and rationalize its behavior during formulation and manufacturing.^16^ Here, we describe a new tool that builds on this approach. Incorporated into CCDC’s Mercury^17^ and CSD Python API software, these surface analysis methods allow for qualitative and quantitative analysis of important properties such as surface chemistry and topology. Recent studies have shown links between key surface properties and punch sticking during tabletting, particularly the presence of polar functional groups on particle surfaces.^18−20^ We therefore seek to demonstrate the applicability of these new surface analysis tools to industrial challenges by examining the specific case of punch sticking during the ibuprofen tablet manufacturing process.

**Figure 1:**
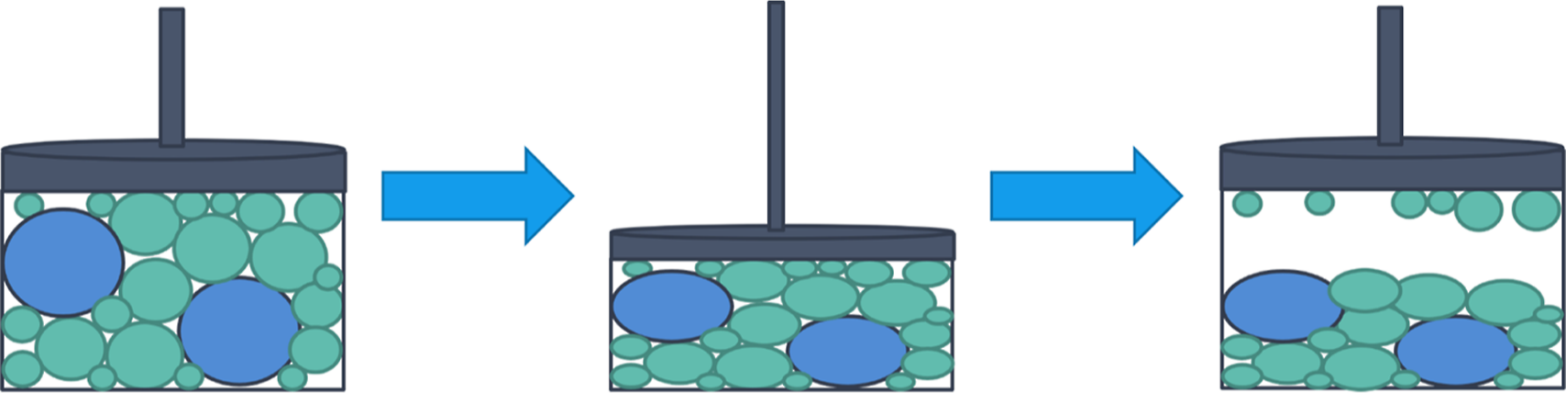
Diagram illustrating punch sticking during tablet compaction. Active ingredient particles (green) can stick to the surfaces of the tablet press (gray) and build up over time.

Punch sticking occurs during tabletting when material adheres to the press, causing inconsistent compaction in subsequent tablets and eventually requiring the operation to be stopped and the process to be taken offline for cleaning and maintenance (Figure 1).^21^ Hooper et al.^22^ found that ibuprofen particles with a lower aspect ratio had a higher sticking propensity. By growing crystals in solvents of different polarities, they were able to modify the shape of the particles, causing them to have a range of different morphologies, from blocks to laths. They calculated a computational representation of the morphologies, using a crystal structure,^23^ Habit98,^24^ and Materials Studio^25^ to obtain the surface energy of each facet. They then measured the sticking propensity of ibuprofen powders to the tablet press and found that an increase in the aspect ratio correlated with a decrease in sticking propensity.

Using a combination of several computational tools and experimental results, including lattice energy calculations and visualization of surface terminations, they were able to conclude that the presence of the carboxylic acid functional group on the surfaces of specific facets caused the powder to have a higher sticking propensity when these specific facets dominated the particle morphology.

Although the particles used for Hooper’s punch sticking study were not face-indexed, and as such, accurately reproducing the morphologies from these results was not possible, work carried out by Cano et al.^26^ determined the growth rates of individual ibuprofen facets in different polar solvents similar to those used in Hooper’s punch sticking study. Using these data allows us to reproduce the observed morphologies for our own calculations using Cano’s measured growth rates.

Currently, carrying out a visual inspection of potentially interesting particle surfaces requires manual effort and chemical intuition to yield qualitative analysis, which is often subjective. The method proposed in this study systematically analyzes surfaces to capture the chemistry, topology, and potential surface activity in a quantitative manner while providing simple-to-understand visualizations for qualitative analysis. This results in an improved approximation of each surface’s contribution to the overall particle properties that can be adjusted based on the observed morphology and the relative ratios of different facets.

## 2 Methodology

To quantify the differences in surface chemistry and topology for a given set of particle shapes, surface representations were constructed from a set of observed Miller planes and the crystallographic structure of polymorph 1 of ibuprofen (CSD Refcode IBPRAC^23^).

The particle shapes used in this study were derived from Cano et al.,^26^ where crystals grown from two different solvents, ethanol and ethyl acetate, gave crystals with different aspect ratios which matched those reported in Hooper et al.^22^

All analyses were carried out using the CSD Python API^27^ and various mathematical (Numpy^28^), plotting (Plotly^29^), and data analysis (Pandas^30^) libraries in Python 3.9.^31^ Surfaces and morphologies were visualized using CCDC’s Mercury 2023.3 software.^17^

### 2.1 Surface Generation

Surfaces were defined using the orientation (Miller Index), offset (distance along the plane normal from the plane origin), and crystal structure.

To reduce the amount of computational effort required to represent a surface, the periodic boundaries of a surface slab were calculated by using the unit cell. The slab was described in 3-dimensions, with **u–v** vectors as in-plane vectors constituting the sides of the surfaces and **w** as the thickness vector.

**Figure 2:**
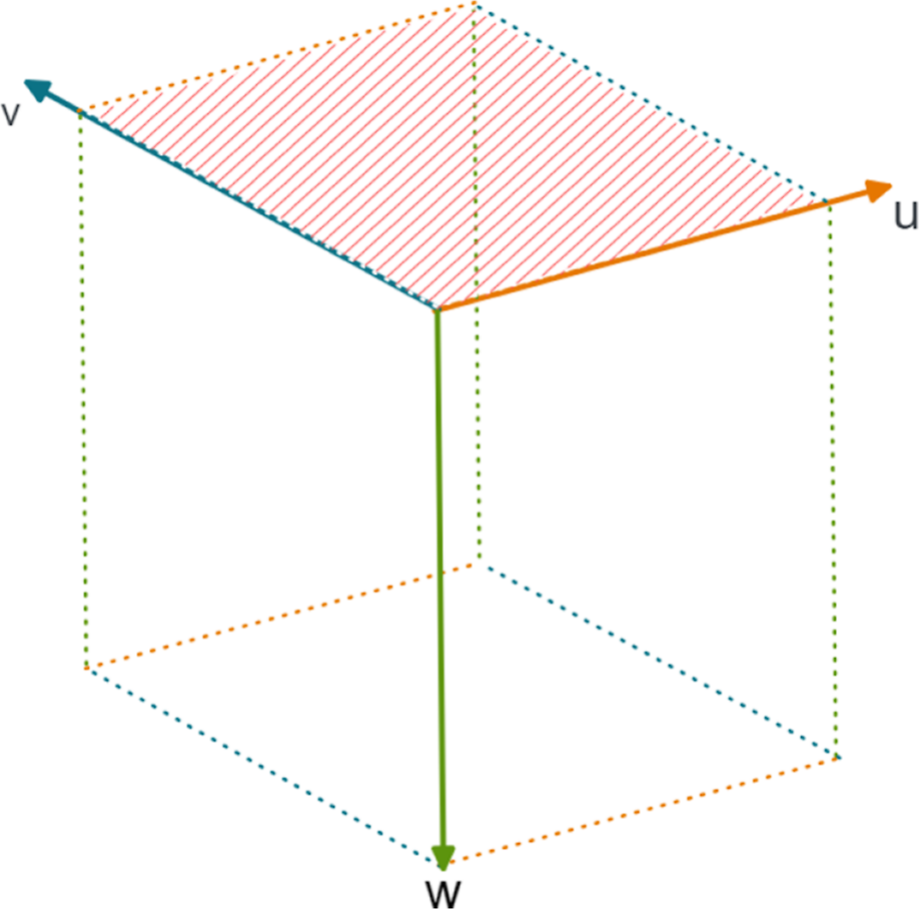
Periodic bounding box view of a defined surface. Periodic in-plane vectors **u** and **v** are shown with orange and blue lines, respectively, and the thickness vector **w** is in green. The surface plane is indicated in red. Dashed lines represent the remaining edges of the bounding box.

The periodic boundary (Figure 2) for the slab is calculated by minimizing the area of the **uv** parallelogram to create the minimum repeating unit. The slab thickness **w** is defined by the unit cell body diagonal. Molecules whose centers of geometry lay within this bounding box were included in the slab representation.

A surface topology was calculated as a mesh of nodes using an algorithm similar to the existing void calculations algorithm from Mercury^32^ to enable analysis of the physical properties of the surface. Briefly, a probe of a given radius is placed on a grid with a defined grid spacing over the slab of molecules defined above. The topology is computed where the probe intersects the van der Waals radii of the surface atoms, resulting in a contact surface as defined by Barbour.^32^ For this study, a probe radius of 1.2 Å and a grid spacing of 0.3 Å were used; these values gave the best balance of surface fidelity with computational speed and are consistent with similar applications of this algorithm. Surface atoms were classified as those that were in contact with the nodes of the surface topology. Contacts were defined by intersecting van der Waals radii between the atom and the surface node, where the surface node was a point in space.

### 2.2 Surface Descriptors

#### 2.2.1 Density of Functional Groups

The densities of hydrogen bond donors, “unsatisfied” hydrogen bond donors, hydrogen bond acceptors, and aromatic bonds were calculated by counting the number of atoms or bonds that contributed to each category per the projected surface area.

Hydrogen bonds were defined using the standard settings in Mercury while requiring a hydrogen atom to be present on the donor atom. Unsatisfied hydrogen bond donors were defined as donors whose hydrogen atom was not participating in a hydrogen bond with another atom in the surface and thus remains “available” for hydrogen bonding.

#### 2.2.2 Surface Polarity and Charge

The topological polar surface area (TPSA) was calculated using the summation of coefficients of the ibuprofen molecular fragments that were on the surface. The coefficient values and fragments used for identification are those from Ertl et al.^33^

Similarly, the total Gasteiger charges (TGC) were computed by summing the Gasteiger charges^34^ of all the atoms present on the surface. TGC values were normalized by the surface area.

#### 2.2.3 Functional Group Detection

Functional groups of ibuprofen molecular fragments were classified by querying the chemistry of the system against the central groups in the IsoStar Interaction Library.^35^ Functional groups identified were then cross-referenced to the list of surface atoms, allowing the identification of surface terminating functional groups.

#### 2.2.4 Surface Roughness

Topological descriptors were calculated to compare the surface roughness, such as rugosity, which capture both 2- and 3-dimensional features of the topology.

The true surface area was defined as the area of the topology, including the depth of the valleys and the height of the peaks. The projected area was computed as the reticular area of the surface as defined by the vectors **u** and **v** described above. The rugosity is given as the ratio between the true and projected surface areas.

Root mean square deviation (rmsd), skewness, and kurtosis were used to describe the different properties related to the surface height deviation from the mean plane. The equations used to calculate these descriptors are given in the Supporting Information.

### 2.3 Full Interaction Maps on the Surface (FIMoS)

By utilizing interaction data derived from the CSD it was possible to search for interactions on the surface based on the functional groups that are present. Full Interaction Maps^36^ are typically used to indicate the probability of interaction between a molecular fragment and a given probe. The existing method was extended to consider the interactions of probes with fragments across the calculated surface.

From the generated surface representation, a grid was placed over the slab where only unique interactions would be accounted for. For the purposes of this study, interactions with a variety of probes were calculated, and the resulting grids were analyzed, to explore a range of chemical environments: uncharged NH nitrogen to represent a generic hydrogen bond donor; carbonyl oxygen to represent a generic hydrogen bond acceptor; water oxygen to represent hydrogen bond donors and acceptors simultaneously; aromatic CH carbon to represent hydrophobic interactions; charged NH nitrogen to represent a charged species. Higher grid densities in Full Interaction Maps indicate a greater probability above random of finding a particular interaction within the CSD. Hotspots were calculated as clustered grid points with a value above a given threshold, and these hotspots were subsequently used to weight count values. Thus, we define the weighted hotspot count as a summation of all hotspot probabilities.

### 2.4 Combining Facet Descriptors for Particle Representations

Using experimental facet growth data measured by Cano et al.,^26^ the morphologies of the particles were computationally reproduced. The facet representation (*F*_r_) of each surface (*A*_*i*_) was calculated by using eq 1
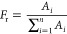
1

Individual descriptors (*D*_*i*_) were normalized by the projected surface area before weighting to the facet representation, as shown in eq 2, where *D*_p_ is the particle descriptor.
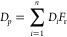
2

### 2.5 Morphology Generation

**Figure 3:**
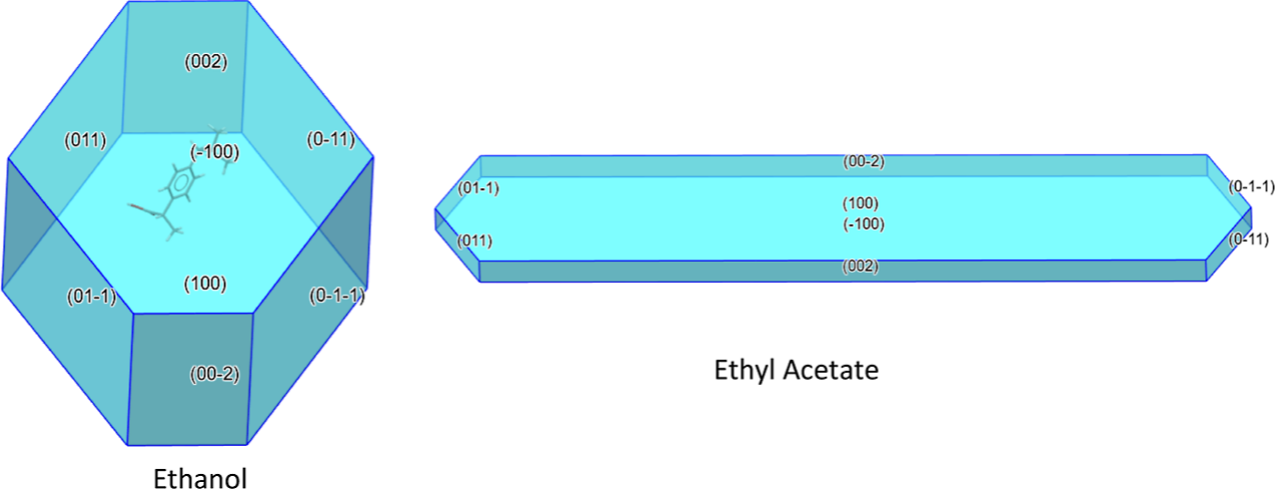
Morphology representations based on facet growth data from Cano et al.^23^

Figure 3 shows the morphologies derived from growth rates measured by Cano et al.^26^ Morphologies were generated by editing morphology .cif files to include the *h*, *k*, *l* values and corresponding growth rates as perpendicular distances for each face. Figure 3 shows that the block shaped crystal grown from ethanol has a lower aspect ratio compared to that of the lath grown from ethyl acetate. Crucially, while the shapes of these particles are different, the same surfaces are expressed, albeit with different ratios. The corresponding percentage area representation of each surface can be found in Table 1.

**Table 1:** Facets and Their Percentage Area Representations for Ibuprofen Crystals Grown from Ethanol and Ethyl Acetate

surface	solvent/shape description	
	ethanol/block (%)	ethyl acetate/lath (%)
{002}	14.22	19.61
{011}	48.77	4.04
{100}	37.01	76.35

The presence of the {002} facets is similar for both the ethanol and ethyl acetate particles, 14.22 and 19.61% of the total surface area, respectively, while the representation of {011} is significantly reduced from 48.77 to 4.04%. As such, the {100} facet is the most dominant surface for the lath-shaped particle grown from ethyl acetate, covering 76.35% of the particle compared to 37.01% for the ethanol (block) morphology.

## 3 Results and Discussion

Having described this new method of surface analysis, we propose how it can be used to predict particle properties from a crystal structure. First, we describe each aspect of the new analysis on the {011} surface of IBPRAC by calculating the chemistry, roughness, and possible surface interactions. We then compare all of the surfaces to one another and describe the average particle properties and how they differ between morphology representations. Finally, we compare our results to those reported by Hooper et al.^22^ and assess the validity of utilizing surface analysis for predicting punch sticking propensity.

### 3.1 Surface Representations

**Figure 4:**
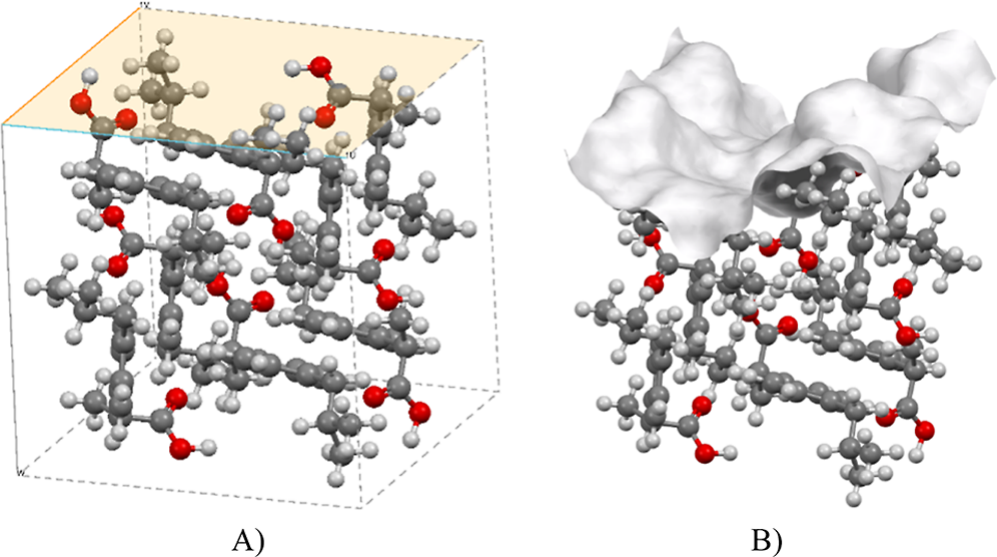
Periodic boundary representation for the {011} facet of IBPRAC with an offset of 0.00 Å. Surface vectors **u** (blue) and **v** (orange) define the span of the surface, with the vector **w** (gray) determining the thickness. (A) Orange plane represents the termination of the surface. (B) Corresponding van der Waals contact surface.

The surface representation is defined by using the unit cell, which encapsulates the repeat unit of a given orientation and offset. The periodic boundary is illustrated in Figure 4A, where a two-dimensional periodicity describes the slab of molecules used to define the surface.

The van der Waals contact surface, as seen in Figure 4B, is used to calculate the surface chemistry and roughness descriptors. Due to the surface being generated using a probe and grid model, the accuracy and smoothing of the surface can change with different probe sizes/grid spacings.

### 3.2 Surface Chemistry

**Figure 5:**
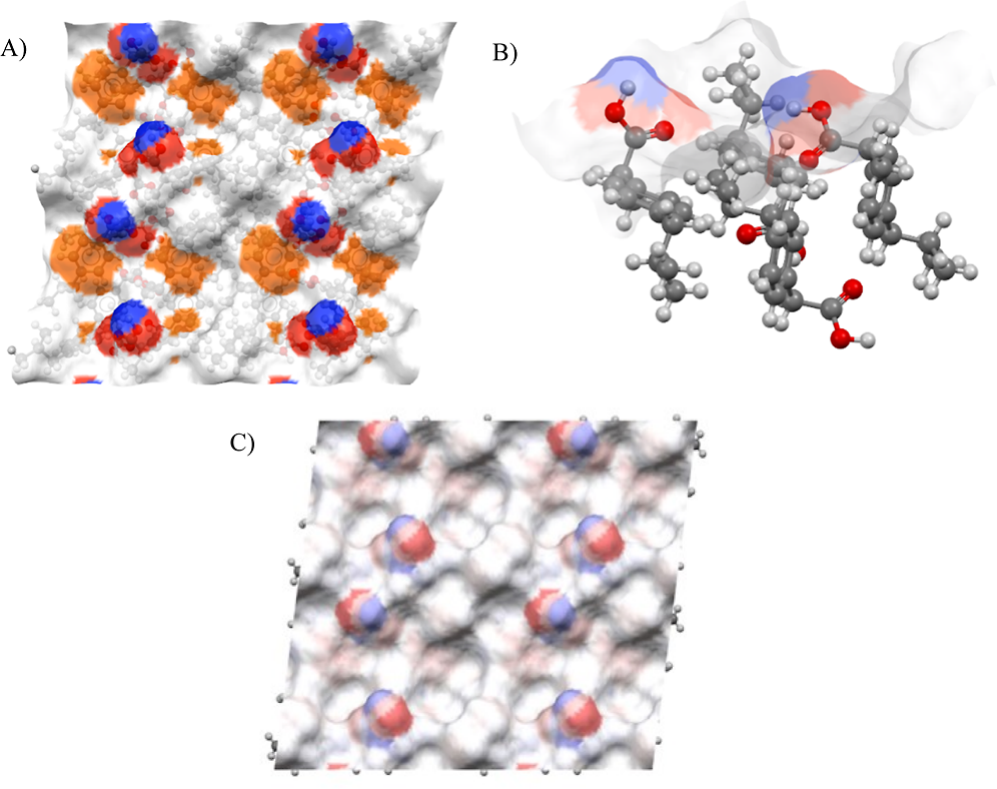
A 2 × 2 representation of the IBPRAC facet {011} showing different surface properties. (A) Location of hydrogen bond donors (red) and acceptors (blue) and aromatic bonds (orange). (B) Close-up of the carboxylic acid group in a 1 × 1 surface representation with overlaid hydrogen bond donor and acceptor colors. (C) Surface charge distribution based on Gasteiger charges (blue positive, red negative).

Surface {011} was calculated to have twice the number of hydrogen bond (HB) acceptors than donors, as 0.021 and 0.010 counts/Å^2^, visualized as the blue regions on the surface in Figure 5A. The HB donors are classified as unsatisfied as their available proton is protruding from the surface and is available for donation. A visualization of the unsatisfied HB donors is shown in Figure S2. The –OH group in the carboxylic acid acts as both a donor and an acceptor, while the carbonyl oxygen acts only as an acceptor, resulting in a 2:1 ratio.

The density of aromatic bonds is greater than the densities of HB donors/acceptors, at 0.083 counts/Å^2^, as the aromatic ring is oriented parallel to the surface plane, and thus all ring bonds are surface-terminating.

The distribution of surface charge is shown in Figure 5C, with the total surface charge calculated as −0.343 and a TPSA of 74.6 Å^2^.

Results from the automatic functional group identification for surface-exposed groups are shown in Table 2.

**Table 2:**
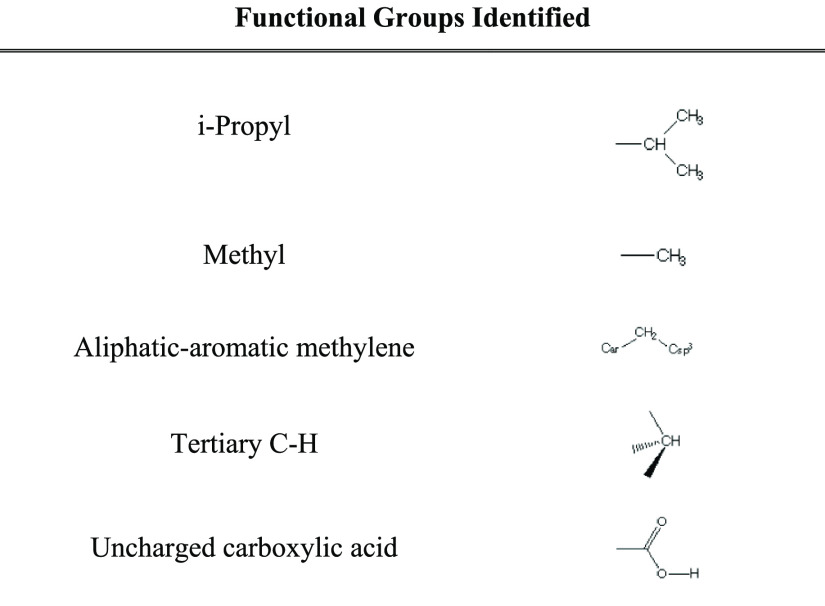
Functional Groups Found on the {011} Surface

The {011} surface contains all of the functional groups identified in the IBPRAC molecule. Most notably, the uncharged carboxylic acid is present on the surface. This supports the previously described densities of hydrogen bond donors and acceptors and confirms observations made by Hooper et al.^22^

### 3.3 Surface Roughness

We quantified the roughness of the individual surfaces by using the nodes describing the surface topology. Roughness is measured on the atomistic scale and assumes periodicity of the surface. Surface roughness is described by the rugosity, rmsd, skewness, and kurtosis; equations for these can be found in Table S1.

**Figure 6:**
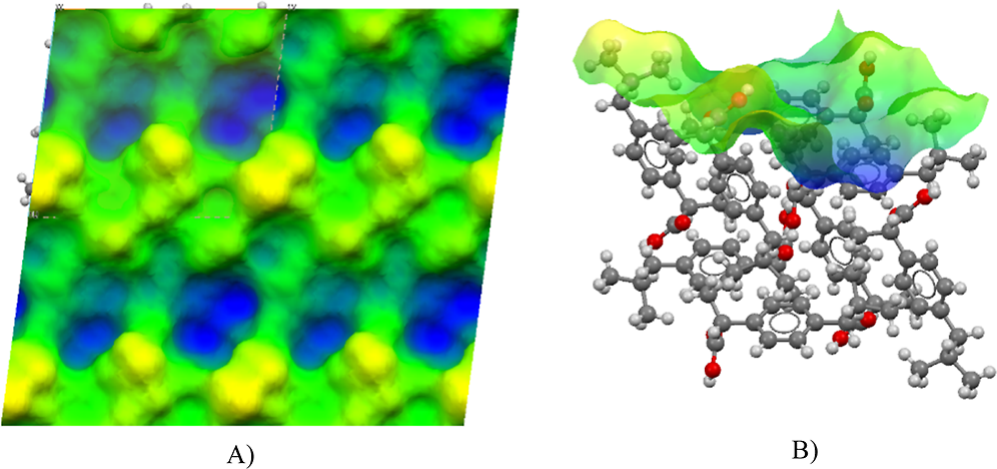
IBPRAC surface {011} colored by topology. (A) Top-down view of the surface topology of a 2 × 2 surface where roughness is illustrated by differences in color (green indicates the height around the mean plane with blue below and yellow above the plane). (B) Side on view of a 1 × 1 surface slab with topology overlaid.

Figure 6 shows the roughness of the IBPRAC {011} surface. The color difference in the topology illustrates a difference in height from the mean plane. Blue colors show the depth of valleys, while yellow is the height of the peaks. Figure 6A shows a top-down view of a 2 × 2 surface where the valleys are visible along with the short peaks.

Rugosity summarizes the roughness of the surface and is the ratio between the projected and true surface area. Surface {011} has a rugosity of 1.462, which can be interpreted as being relatively rough. A perfectly smooth surface would have a rugosity of 1.0. The rmsd of all of the node heights was measured as 1.249 Å.

The skewness and kurtosis numerically describe the observations in Figure 6 that valleys have a slightly greater presence on the {011} surface, with a negative skewness value of −0.054, indicating the height distribution is skewed below the mean plane. Pearson’s kurtosis explains the sharpness of the height distribution where extremely deep valleys/high peaks have a kurtosis greater than 3. In the case of the {011} surface, we calculated kurtosis at 2.174, in agreement with observations seen in Figure 6 where no significant peaks or valleys are present.

### 3.4 Surface Interactions—FIMoS

Functional group positions impact the roughness of the surface as well as the chemistry exposed to incoming species. By combining the presence of the functional groups on the surface alongside the large data set of interaction data derived from the CSD, we demonstrate how this approach can offer an insight into the possible surface interactions that are driven by structural stability and steric hindrance.

Full Interaction Maps^36^ allow us to identify likely interactions between surface chemistries and a list of probes. The data is derived from the CSD and so uses experimental crystal structures, making FIMoS a rapid, informatics-based approach.

**Figure 7:**
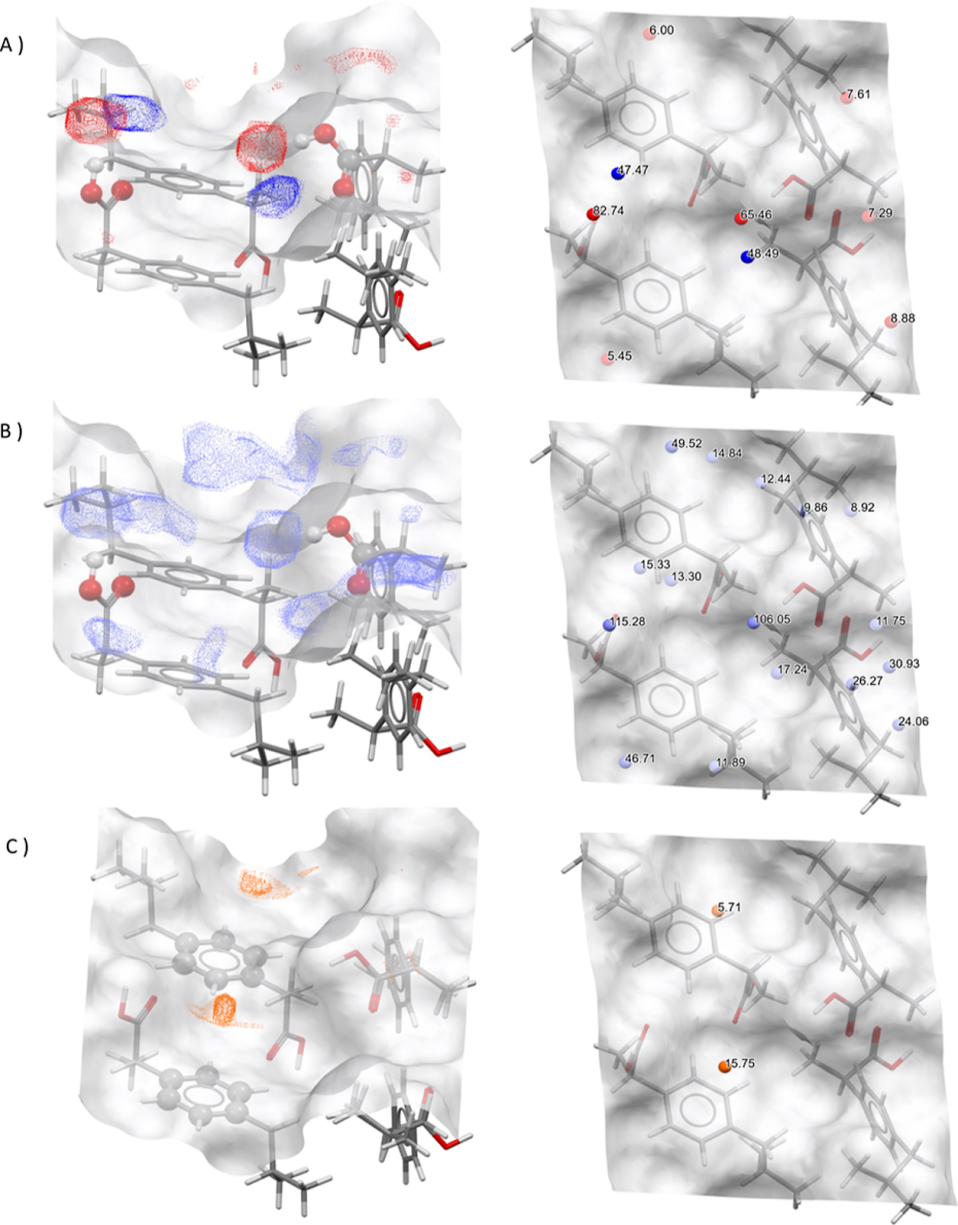
FIMoS data and hotspot positions labeled with probabilities above random (A) for carbonyl oxygen (red) and uncharged NH (blue) probes, (B) Water oxygen probes (light blue), and (C) Aromatic CH (orange).

Analysis of the FIMoS data for uncharged NH probes alongside carbonyl oxygen, as shown in Figure 7A, indicates there is a high probability of interaction with the carboxylic acid group protruding from this surface. The surface area normalized weighted hotspot counts for the two probes were calculated as 0.53 and 1.03, respectively. The carbonyl oxygen probe (red) has a higher probability than the NH probe due to the position of the –OH group on the surface and the availability of this hydroxyl hydrogen atom to be donated, in comparison to the location of the C=O group, which is buried deeper in the surface.

The water oxygen showed the highest weighted hotspot count at 2.63, which is also visible in the prevalence of the probe in Figure 7B, where a propensity for this interaction can be found across the surface. The area around the carboxylic acid group has, as might be expected, the highest hotspot scores of 115 and 106.

These observations for the water oxygen probe highlight an important consideration for this approach. Comparison between probes must be done with care as the probe chemistry will heavily influence the possible types of interactions. This is further demonstrated by the aromatic CH probe, where interaction data are naturally more diffuse and probability values are lower compared to the other probes. Hotspots were calculated as 5 and 15 with the surface normalized weighted hotspot count of 0.25.

As such, we recommend using FIMoS to compare between facets to understand preferential probe affinity. Furthermore, the probability values are not necessarily linearly scaled, as they reflect combined probabilities of functional group contributions. For example, in a situation where two HB donors are available within proximity of an incoming HB acceptor, the probability would be larger than simply the summation of the two independent probabilities.

### 3.5 Surface Comparisons

**Figure 8:**
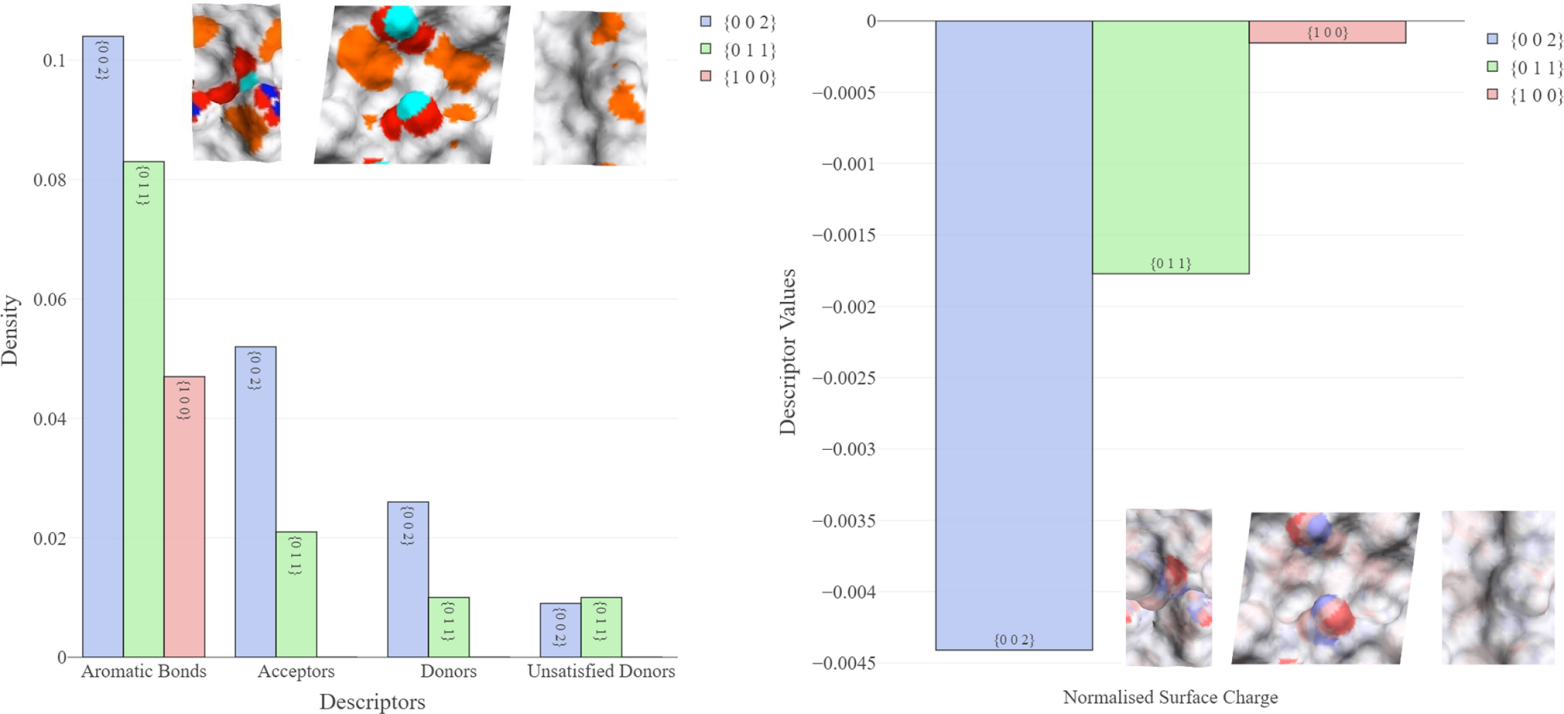
Chemical descriptors across different facets of IBPRAC. (A) Density of aromatic bonds, hydrogen bond donors, acceptors, and unsatisfied donors. (B) Normalized surface charge per unit area.

The density of aromatic bonds and hydrogen bond acceptors, donors, and unsatisfied donors for all surfaces is shown in Figure 8A, indicating a clear difference between facets. The surface topology and chemistry of the three facets are visualized in Figures S3–S5.

As might be expected from the small size of the ibuprofen molecule, all of the facets contain aromatic bonds. We note that {100} contains no donors or acceptors and is therefore expected to be the most chemically inactive surface.

While there exists a difference in hydrogen bond donor/acceptor density values between facets {011} and {002}, {002} shows a larger density of hydrogen bond acceptors. However, some of the hydroxyl groups on the {002} surface are oriented into the bulk, resulting in a density of unsatisfied HB donors lower than that of {011}.

Finally, the normalized surface charge (Figure 8B) indicates that facet {002} is the most charged surface, while {100} is the least charged.

Functional groups listed in Table 3 were located based on the analysis of the surface atoms. The carboxylic acid group, which contains the most electronegative interactions in IBPRAC, was absent from the {100} facet, explaining why this surface is observed to be less active.

**Table 3:** Functional Groups Identified on Surfaces

functional groups identified	surfaces present
i-propyl	{011}, {002}, {100}
methyl	{011}, {002}, {100}
aliphatic–aromatic methylene	{011}, {002}, {100}
tertiary C–H	{011}, {002}, {100}
uncharged carboxylic acid	{011}, {002}

Table 4 shows that in this system the {011} and {100} facets have smoother surfaces with rugosities of 1.462 and 1.374 respectively, while facet {002} is demonstrably rougher with a rugosity of 2.022. The rmsd indicates the difference in height for the peaks and valleys compared to the mean plane, where {011} has a larger rmsd of 1.249 Å compared to {100}. {002} has the largest rmsd of 2.462 Å, further demonstrating its higher roughness.

**Table 4:** Topology Descriptors

facet	rugosity	rmsd	skewness	kurtosis
{011}	1.462	1.249	–0.054	2.174
{002}	2.022	2.462	0.070	2.012
{100}	1.374	0.859	0.006	2.038

The skewness between surfaces agrees with qualitative observations of the surface topology. Surface {002} was dominated by peaks, while {011} shows more valleys, and {100} is relatively flat. The kurtosis was comparable between surfaces, indicating that no extreme valleys or peaks are present.

Based on these observations, it is expected that any morphology dominated by the {011} and {002} facets will be more interactive, compared to any particles where the {100} facet dominates, which are identified to be smoother and more inactive.

**Figure 9:**
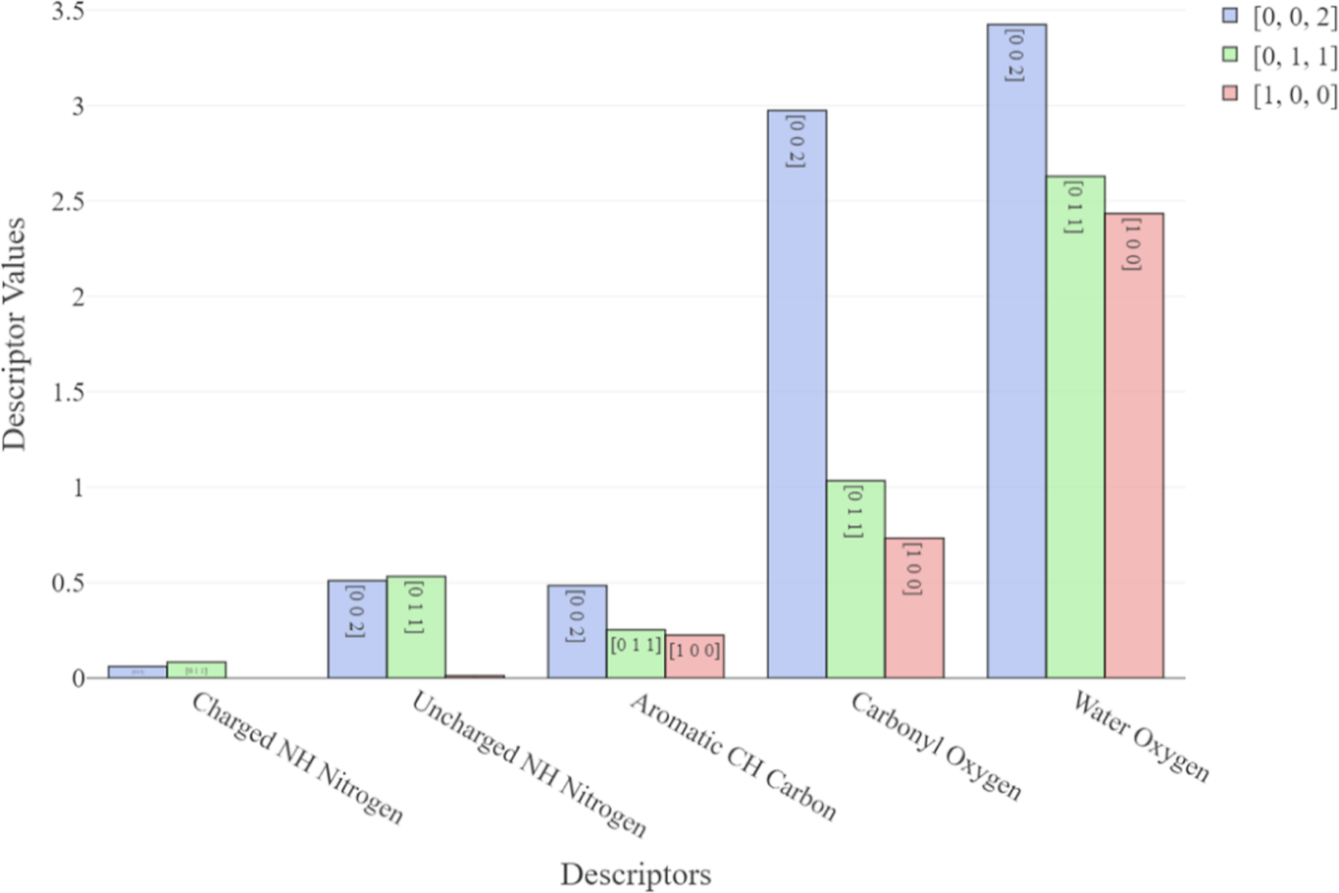
Weighted hotspot counts for different FIMoS probes across all facets.

Figure 9 shows weighted hotspot counts for a variety of different functional group probes across the main IBPRAC surfaces. The {011} surface shows a slightly higher interaction propensity for both the charged and uncharged NH nitrogen probes in comparison to the {002} surface, which shows a markedly higher interaction with the other remaining probes. In all instances, the {100} facet showed the lowest probability of interaction, in line with previous observations that it is likely to be the least chemically active surface.

Combining these individual descriptors, we propose that the {011} and {002} facets would have the most significant effect on particle behavior, although further investigation is required to understand this impact on environmental conditions such as humidity during tabletting. While the {002} appears to have a greater density of negative charge and electronegative groups present, the {011} shows a higher probability of interaction based on the position of the functional groups. Any increase in the percentage representation of these surfaces on the particle will likely impact the powder behavior and likelihood of punch sticking.

### 3.6 Particles

Ibuprofen particles in the Hooper et al.^22^ study showed a higher sticking propensity for low aspect ratio (block) particles compared to those of a higher aspect ratio (lath). They associated the cause of this increase in punch sticking to their observation of an increased presence of carboxylic acid groups on different surfaces, molecular mechanics simulations, and manual partitioning of the functional group energies from a lattice energy calculation. By combining and averaging the surface-specific analysis outlined above across all facets of a given morphology, it becomes possible to automate much of Hooper’s analysis and provide a simple means of comparing two (or more) different morphologies of a given crystal structure.

**Figure 10:**
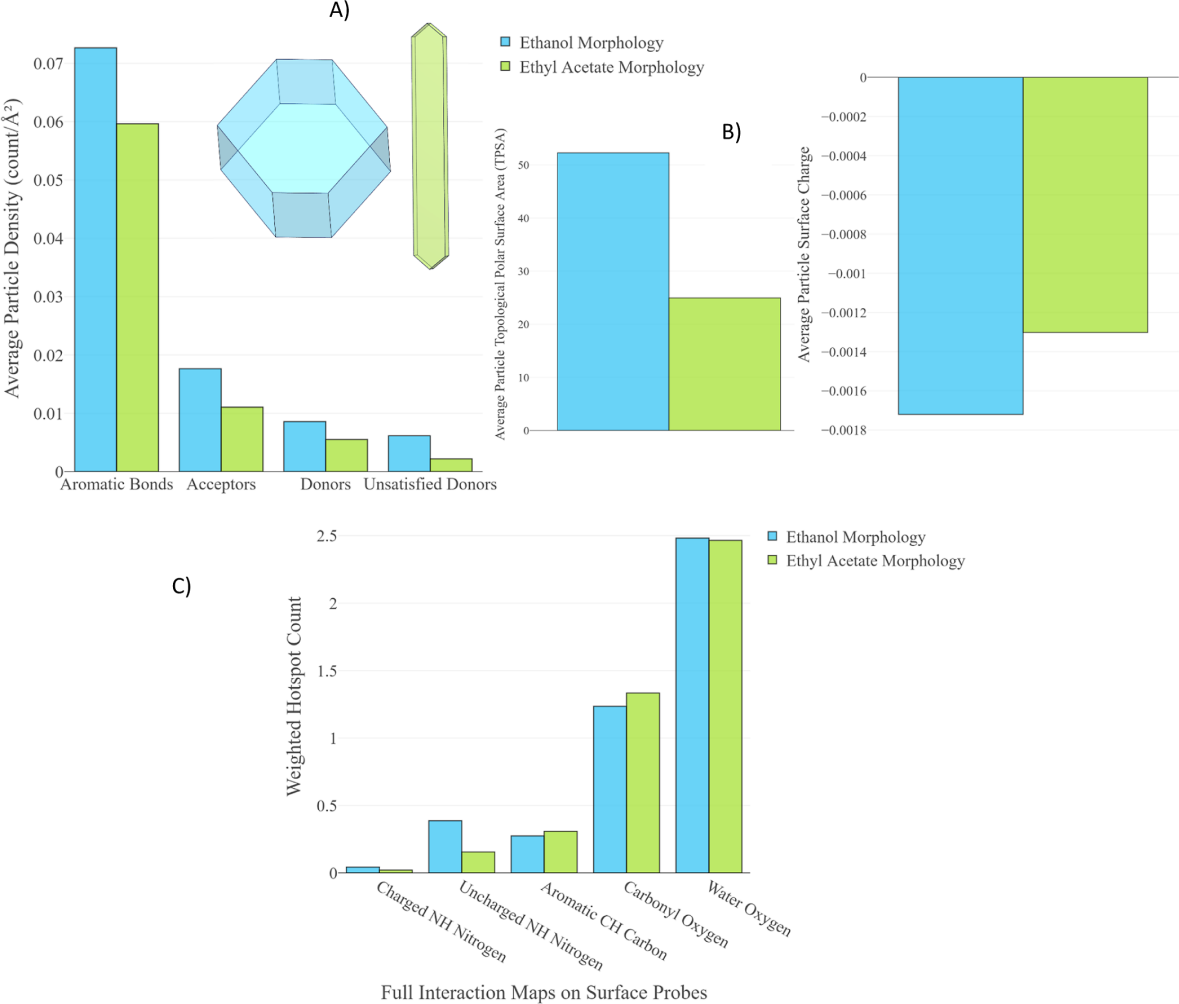
Average particle property data are shown as a representative proportion of each surface. (A) Density of aromatic bonds, hydrogen bond acceptors, donors, and unsatisfied donors. (B) Topological polar surface area (left) and Gasteiger surface charge (right). (C) FIMoS-weighted hotpot count for various probes.

The difference between the two morphologies is shown in Figure 10A. Densities of aromatic bonds, hydrogen bond acceptors, donors, and unsatisfied donors are higher for the more block-like morphologies grown from ethanol (blue). Similarly (Figure 10B), the surface charge and TPSA are higher for the ethanol particles due to the higher proportion of the {011} surface in the ethanol particle. This increase in the polarity, surface charge, and density of hydrogen bonding groups on the ethanol particles compared to those grown from ethyl acetate correlates with the observations made by Hooper et al.,^22^ where more block-like morphologies showed a higher propensity toward punch sticking. In addition to these similar observations for hydrogen bonding surface groups, we also note the increased presence of aromatic bonds on the surfaces of the particles grown from ethanol. While prior work has identified the importance of surface polarity, the role of dispersion interactions during the tableting process should not be overlooked.

The FIMoS interaction data indicate a greater interaction between hydrogen bond donating probes across the particle grown from ethanol. Given the electropositive nature of these probes, this suggests that the block-shaped particles have a higher affinity for positively charged materials. This may lead to an increased tendency for ibuprofen punch sticking when the presence of existing material on the press leads to more hydrogen bonding groups being exposed on the surface.

We agree with Hooper’s observations, noting that the block particle has a higher density of hydrogen bond donor and acceptor groups and shows a higher affinity toward negatively and positively charged FIMoS probes, all of which point to a particle with a higher surface charge and thus a higher likelihood of adhering to a die which is made of metal. Details of the exact material of the die used in the experimental study were not published, and as such, the above claim cannot be verified with observations.

## 4 Conclusions

We present a set of surface analysis tools that enable the visualization and quantification of chemical and topological information derived from crystallographic data. By utilizing functional group analysis, roughness calculations, and interaction informatics, we have demonstrated how this approach can represent surfaces and allow for straightforward comparisons between them.

Facets were described based on chemistry and roughness, utilizing the density of aromatic bonds, hydrogen bond acceptors, donors, and unsatisfied donors, as well as roughness descriptors such as rugosity, rmsd, skewness, and kurtosis. Additionally, we demonstrate the use of Full Interaction Maps to identify regions of preferential interactions for individual probes across the functional groups of a surface.

The sticking propensity of two differently shaped ibuprofen particles reported by Hooper et al. was correlated with surface and particle descriptors calculated from different morphologies derived from a single crystal structure. Our observations agree with those of Hooper and expand on their findings that the presence of a carboxylic acid group on {011} causes the largest difference between the two particles and explains why the block like morphology has a higher electrostatic potential and, consequently, a higher sticking propensity.

While we believe the tool does not replace chemical intuition and expertise, we do believe that it can aid formulators and particle designers in making informed, data-driven decisions to help derisk their formulations. The approach requires only an experimental crystal structure from which to calculate and describe particle surfaces; visualization and analysis of the key properties that can be calculated are possible much earlier in the material discovery process, when quantities of a new chemical are often scarce. As such, we believe that this method provides an efficient computational approach to anticipate potential material property issues downstream much earlier in the development workflow.

## Supporting Information Available

Surface roughness equations used for rmsd, skewness, and kurtosis descriptors, and additional images of surfaces (PDF)
