# Supplementary material for: Surface Analysis—From Crystal Structures to Particle Properties
Source: Cryst Growth Des. 2024 May 1;24(10):4160–9. doi: 10.1021/acs.cgd.4c00259 (PMC11099916; doi:10.1021/acs.cgd.4c00259)
Supplement: Supplementary file 1 — cg4c00259_si_001.pdf [file cg4c00259_si_001.pdf]

Surface Analysis – From Crystal Structures to Particle Properties

Alexandru A. Moldovan and Andrew G. P. Maloney**

The Cambridge Crystallographic Data Centre, 12 Union Road, Cambridge, CB2 1EZ, UK

**amoldovan@ccdc.cam.ac.uk; *maloney@ccdc.cam.ac.uk*

Table S1 - Surface Roughness Equations

Descriptor	Equation
**RMSD**	$\sqrt{\frac{\sum_{i=1}^{n}(x-\bar{x})^2}{n}}$
**Skewness**	$\frac{\frac{1}{n}\sum_{i=0}^{n}(x-\bar{x})^3}{\sigma^3}$
**Kurtosis**	$\frac{\frac{1}{n}\sum_{i=0}^{n}(x-\bar{x})^4}{\sigma^4}$

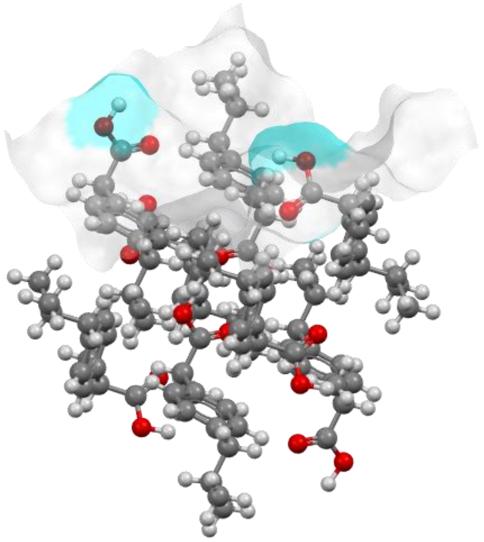

Figure S2 - Surface {011}. The cyan colour represents the position of unsatisfied hydrogen bond donors.

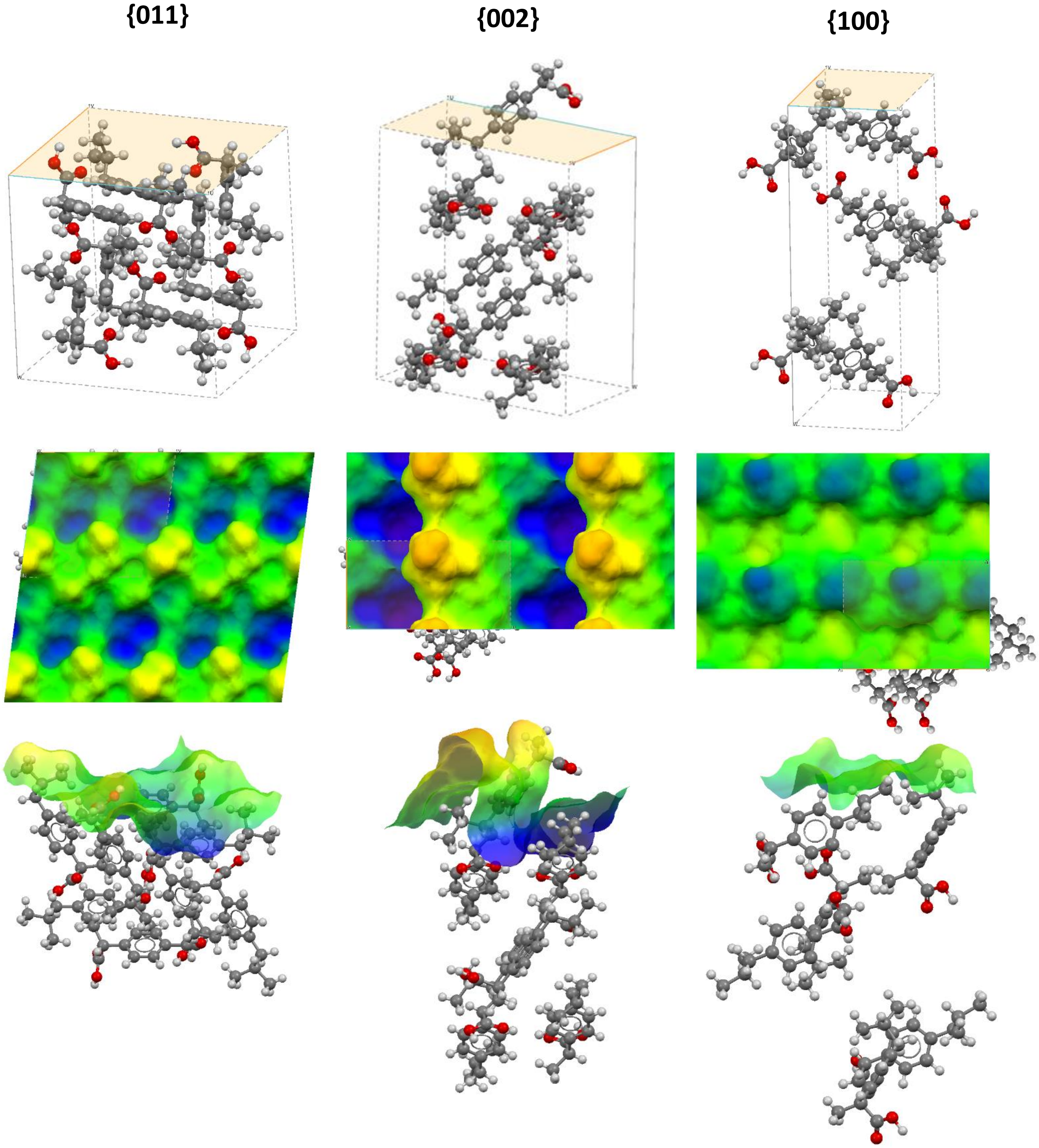

Figure S3 - Three main facets of IBPRAC. *Top* - Slab periodic boundaries with the termination plane being indicated by the orange plane. *Middle* - top-down view of the surface topology of a 2x2 surface where differences in colour illustrate roughness (green indicates height around mean plane with blue below and orange/red above the plane. *Bottom* - Side on view of a 1x1 surface slab with topology overlayed.

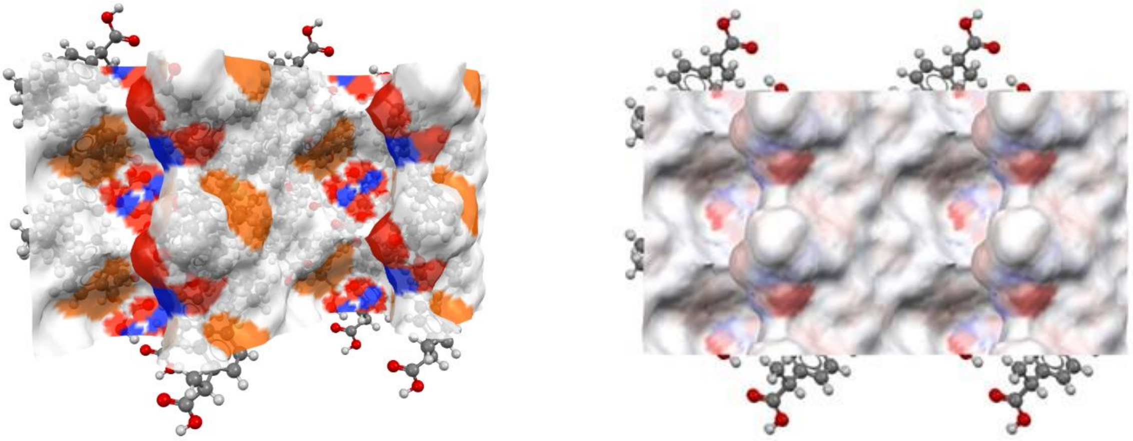

Figure S4 - Surface properties of facet {002}. *Left* - Location of hydrogen bond donors (red) and acceptors (blue) and aromatic bonds (orange). *Right* - Surface charge distribution based on Gasteiger charges (blue positive, red negative).

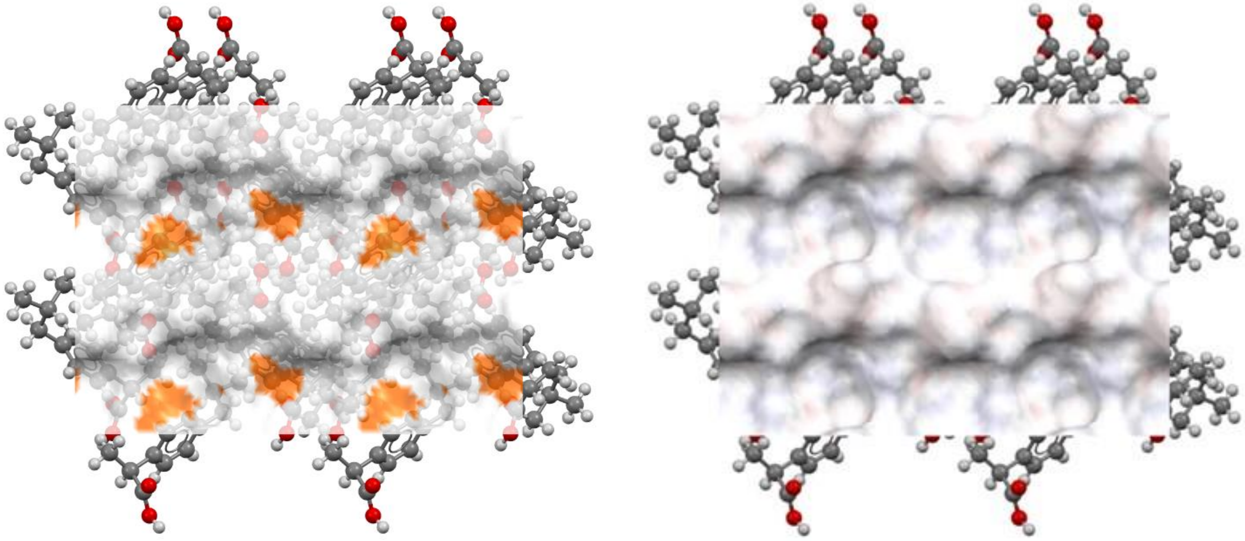

Figure S5 - Surface properties of facet {100}. *Left* - Location of hydrogen bond donors (red) and acceptors (blue) and aromatic bonds (orange). *Right* - Surface charge distribution based on Gasteiger charges (blue positive, red negative).
